# Disparities in Implementing COVID-19 Prevention Strategies in Public Schools, United States, 2021–22 School Year

**DOI:** 10.3201/eid2905.221533

**Published:** 2023-05

**Authors:** Sanjana Pampati, Catherine N. Rasberry, Zach Timpe, Luke McConnell, Shamia Moore, Patricia Spencer, Sarah Lee, Colleen Crittenden Murray, Susan Hocevar Adkins, Sarah Conklin, Xiaoyi Deng, Ronaldo Iachan, Tasneem Tripathi, Lisa C. Barrios

**Affiliations:** Centers for Disease Control and Prevention, Atlanta, Georgia, USA (S. Pampati, C.N. Rasberry, S. Lee, S. Hocevar Adkins, L.C. Barrios);; ICF, Atlanta (Z. Timpe, L. McConnell, C. Crittenden Murray, S. Conklin, X. Deng, R. Iachan, T. Tripathi);; Oak Ridge Institute for Science and Education, Oak Ridge, Tennessee, USA (S. Moore, P. Spencer)

**Keywords:** COVID-19, schools, health equity, coronavirus disease, SARS-CoV-2, severe acute respiratory syndrome coronavirus 2, viruses, respiratory infections, zoonoses, vaccine-preventable diseases

## Abstract

During the COVID-19 pandemic, US schools have been encouraged to take a layered approach to prevention, incorporating multiple strategies to curb transmission of SARS-CoV-2. Using survey data representative of US public K–12 schools (N = 437), we determined prevalence estimates of COVID-19 prevention strategies early in the 2021–22 school year and describe disparities in implementing strategies by school characteristics. Prevalence of prevention strategies ranged from 9.3% (offered COVID-19 screening testing to students and staff) to 95.1% (had a school-based system to report COVID-19 outcomes). Schools with a full-time school nurse or school-based health center had significantly higher odds of implementing several strategies, including those related to COVID-19 vaccination. We identified additional disparities in prevalence of strategies by locale, school level, and poverty. Advancing school health workforce and infrastructure, ensuring schools use available COVID-19 funding effectively, and promoting efforts in schools with the lowest prevalence of infection prevention strategies are needed for pandemic preparedness.

To prevent transmission of SARS-CoV-2, the virus that causes COVID-19, in school settings and maintain in-person learning during the 2021–22 school year, US schools implemented a range of COVID-19 prevention strategies (*1*–*4*). Since the pandemic began, the Centers for Disease Control and Prevention (CDC) has provided guidance for schools on strategies for COVID-19 prevention (*5*). This guidance evolved as new scientific evidence emerged but has consistently emphasized layering multiple prevention strategies. CDC updated guidance for COVID-19 prevention in schools in May 2022 (*5*); recommended core prevention strategies for schools included staying home when sick; optimizing ventilation; practicing proper hand hygiene and respiratory etiquette; performing cleaning and disinfection; and encouraging families, staff, and students to stay up to date on vaccines. Those core prevention strategies are important in preventing the spread of multiple infectious diseases. On the basis of local COVID-19 context, additional prevention strategies included mask requirements, COVID-19 screening and diagnostic testing, cohorting, ventilation improvements, case investigation and contact tracing, and quarantining. Many of the same strategies included in CDC guidance from May 2022 were also included in CDC guidance from August 2021 (*6*), when we conducted our study, and in COVID-19 guidance for safe schools from the American Academy of Pediatrics (AAP) in July 2021 (*7*), which included promoting vaccines, improving ventilation, testing, and cleaning.

Recommended infection prevention strategies varied in terms of expertise, staffing, infrastructure, and financial costs required for implementation. Delivery of specific health services in school settings (e.g., vaccines and tests) might require personnel with medical and public health expertise and existing infrastructure for offering services. For example, implementation studies of school-based influenza vaccination programs have underscored the importance of dedicated staff and program infrastructure for securing supplies and necessary funding, disseminating materials (e.g., consent forms), communicating about the program to students and families, and managing logistics (*8*). Numerous other studies on school-based delivery of vaccines and COVID-19 tests also highlight the value of workforce capacity and infrastructure (*9*–*14*). Furthermore, staff shortages and gaps in expertise within schools might interplay with urban–rural disparities. One study in New Mexico found that nurses in rural schools were more likely to serve multiple campuses, more likely to have fewer years of formal education, and less likely to have continuing education in specific health topics (e.g., anaphylaxis) (*15*). Infection prevention strategies unrelated to health services might also vary by school characteristics. Ventilation improvement strategies, particularly more costly strategies such as upgrading heating, ventilation, and air conditioning (HVAC) systems, might vary by school poverty level. Household studies have found indoor environmental exposures are more concentrated in low-income households, partially because of inadequate ventilation and low air exchange rates (*16*). Evidence on disparities in infection prevention strategies has primarily focused on a single state or school district and a single prevention strategy or a narrow set of prevention strategies. Little is known about how implementing a comprehensive set of infection prevention strategies varies across kindergarten through 12th grade (K–12) schools in the United States by school characteristics. Such findings can guide interventions to improve schools’ ability to prevent transmission of infectious diseases, identify schools to prioritize in resource allocation and capacity building to reduce disparities, and contribute to current and future emergency preparedness.

Accordingly, our study aimed to describe implementing infection prevention strategies relating to vaccines, ventilation, cleaning and disinfection, mask requirements, COVID-19 screening and diagnostic testing, cohorting, case investigation, contact tracing, and quarantining among a nationally representative sample of K–12 public schools early in the 2021–22 school year. The study period coincided with the surge of the of the SARS-CoV-2 Delta variant and was one of high community transmission nationwide, necessitating that all US schools incorporate layered COVID-19 prevention strategies. Further, we characterize disparities in implementation by school level, poverty, urban or rural classification, and presence of health personnel and infrastructure.

## Methods

### Data

The National School COVID-19 Prevention Study (NSCPS) was initiated to better understand implementation and effectiveness of infection prevention strategies in K–12 school settings (*17*,*18*). NSCPS is a population-based, longitudinal study designed to be representative of K–12 public schools in the United States. The study used a single-stage, stratified random sample of K–12 public schools based on strata defined by region (Northeast, South, Midwest, or West), school level (elementary, middle, or high), and National Center for Education Statistics (NCES) locale (city, town, suburb, or rural). School locale was categorized on the basis of the NCES locale classification scheme, derived from the US Census Bureau’s standard urban and rural definitions, which are based on population size and proximity to populated areas (*19*). The allocation was nearly proportional to ensure approximately equal probabilities for schools, which is an efficient design for a survey in which schools (rather than students) are the unit of analysis.

The sampling frame for this study consisted of public K–12 schools. We excluded the following school types: private schools, alternative schools, schools providing special services to a pull-out population enrolled at another eligible school, schools run by the US Department of Defense, and schools with <30 students. We followed the cohort of schools for 5 waves of data collection from June 2021 through May 2022. For each wave, a school-level designee was invited to complete a survey on COVID-19 prevention strategies and COVID-19–related outcomes.

We report data from a cross-sectional analysis of wave 2, the first wave of the 2021–22 school year. The wave 2 survey was administered during October 5–November 19, 2021, and included 81 survey questions primarily assessing the implementation of COVID-19 prevention strategies. A draft version of the survey was pilot-tested with a small number of school principals (n = 8), whose feedback was incorporated in the final survey. Each participant was given a unique link to complete the survey online. Of the 1,602 schools invited to participate, 437 (27%) completed the survey. The primary survey respondents were principals (n = 340, unweighted) and school nurses (n = 39, unweighted). Respondents were offered an electronic gift card valued at $50 for their time and effort. This study was approved by the Institutional Review Board of ICF, a research and evaluation consulting firm, in accordance with CDC’s policies.

### Measures

We examined 21 school-level prevention strategies (e.g., promoting vaccination) assessed through the survey questions (Appendix Table 1). We obtained 2 school-level characteristics from the survey: having a school-based health center (SBHC) and having a full-time school nurse. We categorized school level as elementary (any grade from kindergarten through grade 4), middle (any grade 7 or 8), or high (any grade from 10 through 12). We did not use grades 5, 6, and 9 to categorize school level, and we considered schools categorized as multiple school levels (e.g., kindergarten through grade 8) to be separate schools for sampling purposes. We linked NSCPS surveys with the MDR database, which provides information about individual US schools (*20*). We used the percentage of students eligible for free or reduced-price meals during the 2019–20 school year as a proxy for school-level poverty (*21*,*22*). High-poverty schools had >76% of students eligible for free or reduced-price meals, mid-poverty schools had 26%–75% eligible, and low-poverty schools had <25% eligible (*23*). We categorized school locale according to the NCES locale classification scheme (town, suburb, rural, or city) (*19*). To capture local COVID-19 dynamics preceding survey administration, we pulled from CDC’s county-level community transmission level data the total number of new cases per 100,000 persons within the previous 7 days in each school’s county on September 23, 2021 (i.e., 2 weeks before the survey opened).

### Statistical Analyses

We accounted for survey nonresponse by creating survey weights. Examined school characteristics were not significantly associated with participation except for school affluence level, a measure in MDR’s database summarizing the socioeconomic status of a school derived through a proprietary algorithm (*20*). Schools that were low or below average in affluence were more likely to participate than schools that were average, above average, or high in affluence; thus, we used school affluence to develop nonresponse adjustment classes (Appendix Table 2). We calculated the weighted prevalence of each prevention strategy and 95% CIs for the overall sample. We also calculated unweighted numbers, weighted prevalence, and 95% CIs of strategies by school-level characteristics and used χ^2^ tests to identify differences. We ran separate weighted logistic regression models with each COVID-19 prevention strategy as the dependent variable and school-level characteristics (i.e., school level, NCES locale, school poverty, having a full-time school nurse, and having an SBHC) as the independent variables, controlling for new cases per 100,000 persons in the previous 7 days in the county. We selected independent and control variables on the basis of a review of literature on factors influencing implementation of infection prevention strategies in schools; selected controls satisfied criteria for confounder selection (*24*). We calculated adjusted odds ratios (aORs) and defined differences with p values <0.05 as statistically significant. We conducted analyses in R 4.1.2 (The R Foundation for Statistical Computing, https://www.r-project.org) by using the survey package (*25*).

## Results

Participating schools were heterogenous in terms of school level, urban status, size, and the racial composition (Appendix Table 2). Most schools reported having had a school-based system to report COVID-19 outcomes (95.1% [95% CI 92.5%–96.8%]), had a COVID-19 isolation space in school (92.5% [95% CI 89.4%–94.7%]), quarantined students identified as close contacts (83.5% [95% CI 79.3%–87.0%]), adhered to at least daily or between-use cleaning schedules (79.7% [95% CI 75.5%–83.4%]), inspected and validated existing HVAC systems (74.6% [95% CI 69.8%–78.8%]), and maintained a physical distance of >3 feet in classrooms (74.3% [95% CI 69.8%–78.4%]) (Table). In addition, more than two thirds of schools offered COVID-19 diagnostic testing to students and staff (68.7% [95% CI 63.8%–73.3%]) and opened windows when safe to do so (66.8% [95% CI 62.2%–71.1%]). Approximately two thirds of schools required masks for students and staff (66.4% [95% CI 61.9%–70.6%]). Less than one third of schools reported having offered COVID-19 screening testing to students and staff (9.3% [95% CI 6.9%–12.5%]), installed or used high-efficiency particulate air (HEPA) filtration systems in classrooms (27.3% [95% CI 23.3%–31.7%]), and provided COVID-19 vaccines on-campus to staff, students, or their families (30.9% [95% CI 26.5%–35.8%]).

**Table:** Prevalence of COVID-19 prevention strategies implemented by K-12 public schools (N = 437) and school-level characteristics, National School COVID-19 Prevention Study, United States, October 5‒November 19, 2021*

Characteristic	No.	% (95% CI)
COVID-19 prevention strategy†		
Required masks for students and staff		
Yes	263	66.4 (61.9–70.6)
No	139	33.6 (29.4–38.1)
Inspected and validated existing HVAC systems		
Yes	318	74.6 (69.8–78.8)
No	40	10.4 (7.5–14.1)
Don’t know	60	15.1 (11.7–19.2)
Replaced or upgraded HVAC		
Yes	161	39.1 (34.3–44.0)
No	176	41.6 (36.6–46.7)
Don’t know	81	19.4 (15.6–23.8)
Installed or used HEPA filtration systems in classrooms		
Yes	121	27.3 (23.3–31.7)
No	249	60.6 (55.6–65.3)
Don’t know	47	12.2 (9.1–16.1)
Opened doors when safe to do so		
Yes	278	65.9 (61.2–70.2)
No	127	30.9 (26.6–35.4)
Don’t know	13	3.3 (1.8–5.8)
Opened windows when safe to do so		
Yes	283	66.8 (62.2–71.1)
No	120	29.2 (25.1–33.6)
Don’t know	14	4.0 (2.4–6.8)
Adhered to at least to daily or between use cleaning schedules		
Yes	331	79.7 (75.5–83.4)
No	89	20.3 (16.6–24.5)
Maintained physical distance in classrooms		
>3 feet	310	74.3 (69.8–78.4)
No physical distancing requirements or <3 feet	110	25.7 (21.6–30.2)
Had a school-based system to report COVID-19 outcomes		
Yes	395	95.1 (92.5–96.8)
No or don’t know	22	4.9 (3.2–7.5)
Had a COVID-19 isolation space in school		
Yes	383	92.5 (89.4–94.7)
No	32	7.1 (5.0–10.2)
Don’t know	‡	‡
Offered COVID-19 diagnostic testing to students and staff		
Yes	293	68.7 (63.8–73.3)
No	124	31.3 (26.7–36.2)
Offered COVID-19 screening testing to students and staff		
Yes	40	9.3 (6.9–12.5)
No	377	90.7 (87.5–93.1)
Conducted contact tracing		
Yes	228	53.4 (48.5–58.3)
No	167	41.5 (36.7–46.5)
Don’t know	22	5.1 (3.3–7.8)
Quarantined students identified as close contacts		
Yes	322	83.5 (79.3–87.0)
No	65	16.5 (13.0–20.7)
Provided information on COVID-19 vaccines to parents		
Yes	275	65.8 (60.8–70.5)
No	125	30.4 (25.9–35.4)
Don’t know	15	3.8 (2.2–6.3)
Provided information on COVID-19 vaccines to students		
Yes	183	43.6 (38.9–48.4)
No	217	52.7 (47.8–57.6)
Don’t know	15	3.7 (2.2–6.2)
Provided parents or students with information about catching up on missed healthcare (e.g., routine vaccines)		
Yes	215	52.3 (47.0–57.6)
No	159	38.2 (33.2–43.5)
Don’t know	41	9.5 (7.0–12.8)
Provided COVID-19 vaccines on-campus to staff, students, or their families		
Yes	124	30.9 (26.5–35.8)
No	284	67.6 (62.8–72.2)
Don’t know	‡	‡
Provided COVID-19 vaccines through school district events to staff, students, or their families		
Yes	219	53.0 (48.0–58.0)
No	196	47.0 (42.0–52.0)
Tracked vaccination status of students		
Yes	139	33.7 (29.1–38.6)
No	276	66.3 (61.4–70.9)
Tracked vaccination status of staff		
Yes	247	59.9 (55.0–64.6)
No	168	40.1 (35.4–45.0)
School-level characteristics		
School level		
Elementary	236	52.4 (51.1–53.7)
Middle	108	25.7 (24.6–26.8)
High	93	21.9 (20.8–23.0)
NCES locale		
City	117	29.4 (27.3–31.6)
Suburb	121	32.3 (29.9–34.7)
Town	55	12.9 (11.6–14.2)
Rural	102	25.4 (23.4–27.5)
School-level poverty		
Low-poverty	77	18.2 (14.7–22.2)
Mid-poverty	227	58.1 (53.0–63.0)
High-poverty	96	23.7 (19.6–28.4)
Full-time school nurse		
Yes	244	60.4 (56.1–64.5)
No	179	39.6 (35.5–43.9)
School-based health center		
Yes	69	17.3 (13.7–21.7)
No	354	82.7 (78.3–86.3)

*Numbers are unweighted. Percentages and 95% CIs are weighted. HEPA, high-efficiency particulate air; HVAC, heating, ventilation, and air conditioning; K–12, kindergarten through 12th grade; NCES, National Center for Education Statistics. †Appendix** Table 1** describes survey questions and operationalization for COVID-19 prevention strategies. ‡Prevalence suppressed because relative SE is >30%.

### School-Level Mask Requirements, Ventilation Improvements, and Cleaning Procedures

Bivariate analysis indicated that, among 7 strategies related to school-level mask requirements, ventilation improvements, and cleaning procedures, none varied by school level, 2 varied by NCES locale, 4 varied by school poverty, and 1 varied by whether the school had a full-time school nurse and SBHC (Appendix Table 3). After adjustment for all examined school-level characteristics and the county COVID-19 case rate, mid-poverty schools had lower odds of having inspected and validated existing HVAC systems (aOR 0.37 [95% CI 0.16–0.84]), used HEPA filtration systems in classrooms (aOR 0.52 [95% CI 0.28–0.96]), and opened windows when safe to do so (aOR 0.48 [95% CI 0.24–0.95]) than did low-poverty schools (Appendix Table 4). Rural schools had lower odds of having installed or used HEPA filtration systems in classrooms (aOR 0.36 [95% CI 0.17–0.76]) than did city schools. However, rural schools had higher odds of having opened doors (aOR 2.08 [95% CI 1.03–4.17]) and opened windows (aOR 4.51 [95% CI 2.11–9.60]) when safe to do so compared with city schools. Town schools had lower odds of having required masks for students and staff (aOR 0.38 [95% CI 0.17–0.85]) than did city schools. Schools with a full-time school nurse had lower odds of having opened doors when safe to do so (aOR 0.57 [95% CI 0.34–0.96]) than did schools without.

### Physical Distancing, Isolation Space, COVID-19 Testing and Screening, Contact Tracing, and Quarantine Protocols

Bivariate analysis indicated that, among 7 strategies relating to physical distancing, isolation space, COVID-19 testing and screening, contact tracing, and quarantine protocols, none varied by school level, NCES locale, school poverty, or having an SBHC, and 2 varied by having a full-time school nurse (Appendix Table 5). After adjustment for all examined school-level characteristics and the county COVID-19 case rate, schools that had a full-time school nurse had higher odds of having quarantined students identified as close contacts (aOR 2.02 [95% CI 1.05–3.91]) than did schools without (Appendix Table 6).

### Promoting and Tracking Vaccination of Students and Staff

Bivariate analysis indicated that, among 7 strategies relating to efforts to promote and track vaccination of students and staff, 3 varied by school level, 2 varied by NCES locale, 3 varied by school poverty, 3 varied by having a full-time school nurse, and 4 varied by having an SBHC (Appendix Table 7). After adjustment for all examined school-level characteristics and the county COVID-19 case rate, compared with high schools, elementary schools had lower odds of having provided information on COVID-19 vaccines to parents (aOR 0.49 [95% CI 0.25–0.97]); provided information on COVID-19 vaccines to students (aOR 0.15 [95% CI 0.08–0.29]); provided COVID-19 vaccines on-campus to staff, students, or their families (aOR 0.47 [95% CI 0.26–0.87]); and tracked vaccination status of students (aOR 0.45 [95% CI 0.24–0.83]) (Appendix Table 8). Compared with high schools, middle schools had lower odds of having provided information on COVID-19 vaccines to students (aOR 0.39 [95% CI 0.20–0.79]); provided COVID-19 vaccines through school district events to staff, students, or their families (aOR 0.44 [95% CI 0.21–0.92]); and tracked vaccination status of staff (aOR 0.44 [95% CI 0.20–0.95]). High-poverty schools had higher odds of having provided information on COVID-19 vaccines to students (aOR 3.88 [95% CI 1.81–8.30]) and provided COVID-19 vaccines through school district events to staff, students, or their families (aOR 2.47 [95% CI 1.23–4.98]) compared with low-poverty schools. Mid-poverty schools had higher odds of having provided parents or students with information about catching up on missed healthcare (e.g., routine vaccines (aOR 1.91 [95% CI 1.06–3.44]) compared with low-poverty schools. Rural schools had lower odds of having provided COVID-19 vaccines through school district events to staff, students, or their families (aOR 0.45 [95% CI 0.23–0.88]) and tracked vaccination status of staff (aOR 0.45 [95% CI 0.23–0.90]) than city schools. Town schools had higher odds of having tracked the vaccination status of students (aOR 3.09 [95% CI 1.36–7.01]) than city schools. Schools that had a full-time school nurse had higher odds of having tracked the vaccination status of students (aOR 1.80 [95% CI 1.07–3.03]) than those that did not. Schools that had an SBHC had higher odds of having provided COVID-19 vaccines on campus to staff, students, or their families (aOR 2.00 [95% CI 1.03–3.89]) and of having provided COVID-19 vaccines through school district events to staff, students, or their families (aOR 2.25 [95% CI 1.18–4.30]) than those that did not.

## Discussion

At the time of our study, guidance from CDC and AAP recommended a layered approach to COVID-19 prevention in schools, incorporating multiple strategies to curb transmission of SARS-CoV-2 and protect students, staff, and families while maintaining in-person learning (*6*,*7*). These approaches were used to varying degrees by schools, as affirmed by our findings. This heterogeneity might be partially attributable to school-level inequities predating the COVID-19 pandemic (e.g., in terms of financial resources, available staff, and school infrastructure) that affect schools’ ability to implement the recommended infection prevention strategies. The findings reflect not only school-based responses to the pandemic but also the expertise and resources required to implement infection prevention and control in schools more broadly.

In general, strategies that were less resource-intensive had greater uptake than those that were more resource-intensive. For example, most schools reported requiring masks for students and staff. In contrast, prevalence was lower for providing COVID-19 screening testing to students and staff or providing COVID-19 vaccines on-campus to staff, students, or their families. Numerous methods to support school-based vaccination and COVID-19 testing have been documented (e.g., partnerships with local health departments, workforce capacity, and communication with parents and students), as have challenges (e.g., staffing shortages, availability of testing supplies, lack of perceived community support, difficulty reporting test results and obtaining consent forms, and low participation) (*9*–*14*). Identifying additional sources of support at school, school district, community, health department, state, and federal levels might strengthen schools’ capacity to respond to public health emergencies.

Several strategies that are recommended regardless of local COVID-19 community levels, according to updates to CDC guidance released in May 2022 (*5*), such as promoting routine vaccines, had low uptake. Differences in COVID-19 vaccination promotion by school level were likely because vaccines were not approved or widely available for most elementary school–aged children at the time. Only half of schools provided parents or students with information about catching up on missed healthcare (e.g., routine vaccines), which is concerning given recent declines in childhood vaccination coverage (*26*–*28*). Schools can play an important role in educating about, linking to, or directly offering vaccines in accordance with local or state policies, including COVID-19 and routine pediatric vaccines, and CDC has resources for schools and community partners to support such efforts (*29*).

Schools with health infrastructure and personnel (i.e., having an SBHC or full-time nurse) were more likely to have certain prevention strategies in place even after adjustment for other school- and county-level characteristics. Schools with an SBHC might be better equipped to respond to public health emergencies and provide certain health services (e.g., vaccines). The National Association of School Nurses and AAP recommend that every school have a full-time school nurse (*30*,*31*). Nurses undergo training in infection prevention and control, serve as liaisons with local health officials, and are well-positioned to develop comprehensive emergency response procedures (*32*,*33*). Our finding that schools with a full-time school nurse were more likely to have several prevention strategies extends a robust body of research that has linked school nurses to health-promoting practices and programs in schools and positive student health- and service-related outcomes (*30*,*34*). In our study, 60.4% of schools had a full-time school nurse, and only 17.3% had an SBHC. The White House’s 2022 National COVID-19 Preparedness Plan explicitly acknowledges investing in the expansion of nurses in schools as a priority (*35*). Such investments in the school nurse workforce, as well as in expanding the health infrastructure of schools, could provide immediate benefits for COVID-19 prevention in schools and also lead to long-term gains in emergency preparedness for schools, as well as positive downstream effects for other student health-related outcomes.

Since March 2020, the federal government has approved billions of dollars in funding to cover pandemic-related costs for K–12 schools through the US Department of Education’s Elementary and Secondary Schools Emergency Relief Fund (*36*), the Governor’s Emergency Education Relief Fund (*37*), the US Department of Health and Humans Services’ Head Start and Child Care American Rescue Plan funds (*38*), and the Epidemiology and Laboratory Capacity for Prevention and Control of Emerging Infectious Diseases Reopening Schools supplement (*39*). Mid-poverty schools had the lowest prevalence of several prevention strategies, including higher-cost strategies to improve ventilation (e.g., HEPA filtration systems in classrooms), compared with low-poverty and high-poverty schools, as noted in a previous NSCPS publication (*17*). One possible hypothesis explaining this pattern is high-poverty schools might have more experience applying for federal funding and might be prioritized by state and local education agencies for these funds. A recent survey found school districts with a higher percentage of free or reduced-price meal eligibility were more likely to use federal COVID-19 funds for ventilation improvements (*40*). Low-poverty schools might have more existing operational and discretionary funds to rely on for implementation of prevention strategies. Taken together, although all schools might benefit from additional support in implementing prevention strategies, mid-poverty schools, in particular, could require more strategic efforts.

One limitation of our study is that the survey assessed presence of prevention strategies but not nuances related to compliance, participation, and fidelity. Second, the reporting of certain prevention strategies might be subject to social-desirability biases, leading to respondents overreporting that certain strategies were in place. Third, the response rate for our survey was low (27%); however, most school-level characteristics were not associated with survey participation based on our nonresponse analysis (Appendix Table 2), and nonresponse weight adjustments were incorporated. Fourth, because of a limited sample, the presence of unmeasured confounders, and minimal clustering at certain levels (e.g., school district level), we were limited in the number and type of controls we could use. For example, state policies (e.g., mask mandates) may have affected schools’ ability to implement specific prevention strategies (e.g., mask requirements). Future studies may benefit from examining various levels of influence (e.g., national, state, and school district), as well as their interplay with school characteristics, on schools’ implementation of infection prevention strategies.

Despite those limitations, our study expands understanding of COVID-19 prevention strategies used by schools during the 2021–22 school year, by using data from a population-based sample drawn to be representative of K–12 public schools in the United States. Our study documents strategy implementation at the school level, as opposed to the school district level. Although COVID-19 prevention policies are likely set at the school district level, variation exists in what schools implement, and measuring implementation at the school-level can better capture what occurred in practice. Because survey administration coincided with the surge of the SARS-CoV-2 Delta variant, the period examined represents one of high community transmission nationally, which necessitated layered prevention strategies in all schools. Moving forward, schools might consider adapting to their local context and monitoring COVID-19 community levels to guide implementation of prevention strategies (*41*). Our findings show variation in the prevalence of strategy implementation, including lower implementation of several strategies that can be more resource-intensive, particularly among mid-poverty schools, and increased implementation for several key strategies among schools with expanded health infrastructure (e.g., having a full-time school nurse, SBHC, or both). Our findings suggest a need to enhance efforts to ensure schools can take advantage of available federal funding for COVID-19 prevention. Advancing the school health workforce and infrastructure across US schools could provide stronger support for pandemic preparedness.

Appendix Additional information about disparities in implementation of COVID-19 prevention strategies in public schools, United States, 2021–22 school year.
