# Supplementary material for: Disparities in Implementing COVID-19 Prevention Strategies in Public Schools, United States, 2021–22 School Year
Source: Emerg Infect Dis. 2023 May;29(5):937–44. doi: 10.3201/eid2905.221533 (PMC10124646; doi:10.3201/eid2905.221533)
Supplement: Appendix — Additional information about disparities in implementation of COVID-19 prevention strategies in public schools, United States, 2021–22 school year. [file 22-1533-Techapp-s1.pdf]

Article DOI: https://doi.org/10.3201/eid2905.221533

Disparities in Implementing COVID-19 Prevention Strategies in Public Schools, United States, 2021–22 School Year

Appendix

Appendix Table 1. Questionnaire items and operationalization for COVID-19 prevention strategies – National School COVID-19 Prevention Study, United States, October 5–November 19, 2021

COVID-19 prevention strategies	NSCPS survey question(s)	Operationalization
Required masks for students and staff	• At the start of the 2021-2022 school year, did your school have a mask requirement? Mark one response.<br>Response options: Yes; No; Not applicable, my school was virtual at the start of the 2021-2022 school year<br>• (Only shown to those who did not say "No" to previous question) For which of the following groups at your school was mask wearing required? Mark one response for each.<br>Response options: All individuals; Only individuals who are not fully vaccinated; No requirement; My school was virtual at the start of the 2021-2022 school year<br>· Students<br>· Teachers and School Staff	Yes (selected mask requirement for all individuals for both students and teachers and school staff) vs. No (No mask requirement or mask requirement only for some groups of students or teachers and school staff)
	Since the start of the COVID-19 pandemic, has your school taken the following steps to increase ventilation or filter/clean air in school? Mark one response for each. Response options: Yes; No; Don't know; Not applicable, my school has been virtual since the start of the pandemic.	
Inspected and validated existing HVAC systems*	· Inspected and validated existing HVAC systems for cleanliness, function, and code-compliant operation	Yes vs. no/don't know
Replaced/upgraded HVAC*	· Replaced/upgraded HVAC systems	Yes vs. no/don't know
	At the start of the 2021-2022 school year, did your school take the following steps to increase ventilation or filter/clean air in school? Mark one response for each.<br>Response options: Yes; No; Don't know; Not applicable, my school has been virtual since the start of the pandemic.	
Installed or used HEPA filtration systems in classrooms*	· Installed or used HEPA filtration systems in classrooms	Yes vs. no/don't know
Opened doors when safe to do so*	· Opened doors to hallway or outside when safe to do so	Yes vs. no/don't know
Opened windows when safe to do so*	· Opened windows when safe to do so	Yes vs. no/don't know
Adhered to at least to daily or between use cleaning schedules	Which of the following prevention strategies related to cleaning are being implemented at your school? Mark all that apply.<br>· Adhering to at least daily or between use cleaning schedules	Yes vs. No
Maintained physical distance in classrooms	At the start of the 2021-2022 school year, for each of the following spaces, what distance between people did your school try to maintain? Mark one response for each.<br>Response options: Less than 3 feet; At least 3 feet but less than 6 feet; 6 feet or more; Space not used; No physical distancing requirements; Not applicable, my school was virtual at the start of the 2021-2022 school year<br>· Classrooms	No physical distancing requirements or less than 3 feet physical distancing vs. 3 feet or more
Had a school-based system to report COVID-19 outcomes	At the start of the 2021-2022 school year, was there a system for parents to self-report to school administration if any of the following are true? Mark one response for each.<br>· Their child has been diagnosed with COVID-19<br>· Their child is waiting for COVID-19 test results<br>· Their child has been exposed to someone with COVID-19 within the last 14 days	Yes to all three options vs. No/don't know to any

COVID-19 prevention strategies	NSCPS survey question(s)	Operationalization
	Response options: Yes; No; Don't know	
Had a COVID-19 isolation space in school*	At the start of the 2021-2022 school year, did your school have a separate space, away from the general population, to isolate individuals who may have exhibited symptoms related to COVID-19? Mark one response.<br>Response options: Yes; No; Don't know	Yes vs. no/don't know
Offered COVID-19 diagnostic testing to students and staff	At the start of the 2021-2022 school year, how was onsite COVID-19 testing used at your school? Mark all that apply.<br>· For symptomatic students (Q1A)<br>· For students identified as close contacts of persons with confirmed or probable COVID-19 (Q1B)<br>· For symptomatic teachers/staff (Q1C)<br>· For teachers/staff identified as close contacts of persons with confirmed or probable COVID-19 (Q1D)<br>· For screening all or a percentage of students (regardless of vaccination status) on a regular basis (Q1E)<br>· For screening all or a percentage of students who are not fully vaccinated on a regular basis (Q1F)<br>· For screening all or a percentage of teachers/staff (regardless of vaccination status) on a regular basis (Q1G)	Yes to COVID-19 diagnostic testing of students (i.e., selected Q1A, Q1B, Q2A, or Q2B) *and* COVID-19 diagnostic testing to staff (i.e., selected Q1C, Q1D, Q2C, or Q2D) vs. No/don't know to COVID-19 diagnostic testing for students or staff
Offered COVID-19 screening testing to students and staff	· For screening all or a percentage of teachers/staff who are not fully vaccinated on a regular basis (Q1H)<br>At the start of the 2021-2022 school year, how was off-site COVID-19 testing used at your school? Mark all that apply.<br>· For symptomatic students (Q2A)<br>· For students identified as close contacts of persons with confirmed or probable COVID-19 (Q2B)<br>· For symptomatic teachers/staff (Q2C)<br>· For teachers/staff identified as close contacts of persons with confirmed or probable COVID-19 (Q2D)<br>· For screening all or a percentage of students (regardless of vaccination status) on a regular basis (Q2E)<br>· For screening all or a percentage of students who are not fully vaccinated on a regular basis (Q2F)<br>· For screening all or a percentage of teachers/staff (regardless of vaccination status) on a regular basis (Q2G)<br>· For screening all or a percentage of teachers/staff who are not fully vaccinated on a regular basis (Q2H)<br>Response options: Yes; No; Don't know	Yes to COVID-19 screening testing of students (i.e., selected Q1E, Q1F, Q2E, or Q2F) *and* COVID-19 screening testing of staff (i.e., selected Q1G, Q1H, Q2G, and Q2H) vs. No/don't know to COVID-19 diagnostic testing for students or staff
Conducted contact tracing*	Since the start of the 2021-2022 school year, has your school conducted (or partnered with another organization to conduct) contact tracing for COVID-19 infected students, teachers, or staff? Mark one response.<br>Response options: Yes; No; Don't know	Yes vs. no/don't know
Quarantined students identified as close contacts	At the start of the 2021-2022 school year, which of the following best described your school's protocols for quarantining students exposed to someone with COVID-19 at school or a school-related activity? Mark all that apply.<br>· All students who are not fully vaccinated and who are identified as close contacts of a COVID-19 case at school or a school-related activity are required to quarantine (i.e., stay at home and not attend school in-person)<br>· All students who are identified as close contacts of a COVID-19 case at school or a school-related activity are required to quarantine (i.e., stay at home and not attend school in-person), regardless of vaccination status	Yes to either vs. No to both
Had a COVID-19 isolation space in school*	At the start of the 2021-2022 school year, did your school have a separate space, away from the general population, to isolate individuals who may have exhibited symptoms related to COVID-19? Mark one response.<br>Response options: Yes; No; Don't know<br>Since the start of the 2021-2022 school year, has your school provided information about COVID-19 vaccinations?<br>Response options: Yes; No; Don't know	Yes vs. no/don't know
Provided information on COVID-19 vaccines to parents*	• Information for parents	Yes vs. no/don't know
Provided information on COVID-19 vaccines to students*	• Information for students	Yes vs. no/don't know

COVID-19 prevention strategies	NSCPS survey question(s)	Operationalization
Provided parents or students with information about catching up on missed healthcare (e.g., routine vaccines) *	Since the start of the 2021-2022 school year, has your school provided parents or students with information about catching up on any healthcare that may have been missed during the pandemic, including well-child visits and routine childhood/adolescent vaccinations?<br>Response options: Yes; No; Don't know	Yes vs. no/don't know
Provided COVID-19 vaccines on-campus*	· Since the start of the 2021-2022 school year, has your school made COVID-19 vaccinations available to school staff, eligible students, or their families on your campus?<br>Response options: Yes; No; Don't know	Yes vs. no/don't know
Provided COVID-19 vaccines through school district events	· Does your school offer COVID-19 vaccines through school or district events (even if not on campus) to each of the following groups? Mark one response for each.<br>• Students<br>• Teachers or school staff<br>• Students' families/caregivers<br>Response options: Yes; No; Don't know	Yes to students, teachers or school staff, or students' families/caregivers vs. no/don't know to students, teachers or school staff, and students' families/caregivers
	For which groups is your school tracking COVID-19 vaccination status? Mark all that apply.	
Tracked vaccination status of students	· Students	Yes vs. No
Tracked vaccination status of teachers and other school staff	· Teachers and other school staff	Yes vs. No

HEPA = high-efficiency particulate air, HVAC = heating, ventilation, and air conditioning
*For descriptive purposes, the "don't know" category is presented as a separate category in Table (https://wwwnc.cdc.gov/EID/article/29/5/22-1533-T1.htm). For subsequent models, the "don't know" category is combined with the "no" category.

Appendix Table 2. School participation rates by school characteristics – National School COVID-19 Prevention Study, United States, October 7–November 19, 2021

School characteristic	Levels	Sampled Schools	Participating Schools	Non-Participating Schools	Response Rate	Chi-Square P-Value
School Level	Elementary	833	236	597	28.3%	0.98
	Middle	411	108	303	26.3%	
	High	358	93	265	26.0%	
Census Region	Northeast	260	69	191	26.5%	0.12
	Midwest	402	120	282	29.9%	
	South	551	132	419	24.0%	
	West	389	116	273	29.8%	
NCES Locale*	City	415	101	314	24.3%	0.42
	Suburb	462	129	333	27.9%	
	Town	179	57	122	31.8%	
	Rural	437	119	318	27.2%	
	Missing	109	31	78	28.4%	
Urban Status†	No	962	268	694	27.9%	0.52
	Yes	640	169	471	26.4%	
City‡	City	445	109	336	24.5%	0.12
	Non-City	1,157	328	829	28.3%	
School Size§	Large	1,035	266	769	25.7%	0.06
	Small	567	171	396	30.2%	
Affluence¶	Low/Below Avg	630	189	441	30.0%	0.05
	Avg/Above Avg/High	972	248	724	25.5%	
% ELL (English-limited)	Below median	789	218	571	27.6%	0.76
	Above median	813	219	594	26.9%	
Majority White	No	735	187	548	25.4%	0.13
	Yes	867	250	617	28.8%	
School % Black	Below median	802	230	572	28.7%	0.21
	Above median	800	207	593	25.9%	
School % Hispanic	Below median	801	232	569	29.0%	0.13
	Above median	801	205	596	25.6%	
School % Asian	Below median	803	219	584	27.3%	0.99
	Above median	799	218	581	27.3%	
Title 1#	<$150	370	104	266	28.1%	0.68
	≥$150	1,232	333	899	27.0%	
Poverty Level**	Below median	713	205	508	28.8%	0.24
	Above median	889	232	657	26.1%	
AIM Per Pupil Expenditure††	Below median	799	221	578	27.7%	0.73
	Above median	803	216	587	26.9%	

School characteristic	Levels	Sampled Schools	Participating Schools	Non-Participating Schools	Response Rate	Chi-Square P-Value
Current Per Pupil Expenditure[‡‡]	Below median	694	191	503	27.5%	0.85
	Above median	908	246	662	27.1%	
AP Offered[§§]	No	1,337	371	966	27.7%	0.32
	Yes	350	66	199	24.9%	
Student Computer Ratio[¶¶]	Below median	617	173	444	28.0%	0.59
	Above median	985	264	721	26.8%	
Student Teacher Ratio[##]	Below median	694	188	506	27.1%	0.88
	Above median	908	249	659	27.4%	
Before/After school care***	No	1,252	343	909	27.4%	0.84
	Yes	350	94	256	26.9%	
% College bound[†††]	Below median	778	201	577	25.8%	0.21
	Above median	824	236	588	28.6%	
Career and Technical Ed Offered[‡‡‡]	No	1,237	348	889	28.1%	0.16
	Yes	365	89	276	24.4%	
Change in enrollment[§§§]	Decrease	629	179	450	28.5%	0.10
	No Change	425	99	326	23.3%	
	Increase	548	159	389	29.0%	
Library/Media Center[¶¶¶]	No	312	81	231	26.0%	0.56
	Yes	1,290	356	934	27.6%	
Lunch Program[###]	Below median	807	228	579	28.3%	0.38
	Above median	795	209	586	26.3%	
Special Education****	Below median	638	171	467	26.8%	0.73
	Above median	964	266	698	27.6%	

Using data from the sampling frame and other extant data sources (MDR database), the association between school-level characteristics and participation in the study was modelled, as shown in the table above. We developed nonresponse adjustment classes based on the dichotomous affluence level variable which was found to be significantly associated with participation. The nonresponse weight adjustment starts with the school sampling weight (W). The adjustment was performed within a weighting class (k) defined by whether the school was indicated as low/below average affluence or average/above average/high affluence. Within each weighting class, the adjustment was computed as the ratio of two sums of weights: (1) the sum of the school sampling weights over all the sample schools; (2) the sum of these weights for the 437 participating schools. Post-stratification cells were based on the design strata. For schools in each stratum, the post-stratification weight adjustments were calculated as the total number of schools in the frame for that post-stratum divided by the sum of the product of the nonresponse adjusted weights over all participating schools in the post-stratum. The final weights were the result of the post-stratification adjustments. For each post-stratum (jk), the weights can be expressed as the following product of the post-stratification and nonresponse adjustments and the school sampling weights. Final survey weights incorporated these nonresponse adjustment classes, as well post-stratification based on the design strata, and were used in all analyses.

$$W_{NRjk} = \frac{\sum j\varepsilon S\ W_{jk}}{\sum j\varepsilon P\ W_{jk}}$$

$$W_{Final\ jk} = W_{jk} * W_{NRjk} * W_{PSjk}$$

*The National Center for Education Statistics (NCES) locale classifications categorizes the area where schools are located based on the U.S. Census Bureau’s standard urban and rural designations.

[†]Urban status is an alternate categorization of the NCES locale classification, such that “no” includes suburbs and rural locales and “yes” includes city and town locales.

[‡]City is an alternate categorization of the NCES locale classification, such that “city” includes city areas and “non-city” includes suburb, town, and rural areas.

[§]Small schools contained fewer than 28 students at any grade level and large schools contained greater than or equal to 28 students at any grade level.

[¶]The Affluence Indicator uses a proprietary algorithm developed to rank the socioeconomic status of a school.

[#]Schools that allocated greater than or equal to $150 of Title 1 funding per student were categorized as “≥$150” and schools that allocated less than $150 of Title 1 funding per student were categorized as “<$150”.

**Poverty level data is sourced from the U.S. Census Bureau’s Small Area Income and Poverty Estimates (SAIPE) program. Median poverty is determined by a formula (Orshansky Indicator) based on family income and size.

[††]AIM Per Pupil Expenditure refers to the total dollar amount of instructional material expenditures. The per student data is determined by dividing the whole dollar for all instructional materials expenditures by district enrollment.

[‡‡]Current Per Pupil Expenditure represents the total operating cost for the district per student, including teacher salaries, instruction, support service, and food service. The per student data is determined by dividing the whole dollar current expenditures by district enrollment.

[§§]AP Offered refers to advanced placement courses offered within schools.

[¶¶]Student Computer Ratio refers to total computers in a school per enrolled student.

[##]Student Teacher Ratio is the total number of students per teacher within a school.

***Before/After School Care refers to schools that provide care to students outside of school hours.

[†††]% College bound is the percentage of 2- or 4-year college bound 12th grade students. Data is applied to all schools within a district.

[‡‡‡]Career and Technical Ed Offered refers to schools within districts providing a broad scope of vocational subjects.

[§§§]Change in enrollment describes the change in school or district enrollment from the previous year.

[¶¶¶]Library/Media Center refers to schools with a formal library or media center.

[###]Lunch Program is the percentage of students that are enrolled in a free/reduced price lunch (FRPL) program using 2020 MDR data.

****Special Education identifies institutions that provide special education classes to children with special needs.

Appendix Table 3. Prevalences and unadjusted associations between school-level characteristics and school-level mask requirements, ventilation improvements, and cleaning procedures – National School COVID-19 Prevention Study, United States, October 5–November 19, 2021

	Required masks for students and staff		Inspected and validated existing HVAC systems		Replaced/upgraded HVAC		Installed or used HEPA filtration systems in classrooms		Opened doors when safe to do so		Opened windows when safe to do so		Adhered to at least to daily or between use cleaning schedules	
Characteristic	n*	% (95% CI)†	n*	% (95% CI)†	n*	% (95% CI)†	n*	% (95% CI)†	n*	% (95% CI)†	n*	% (95% CI)†	n*	% (95% CI)†
School level														
Elementary (n=236)	139	64.7 (58.5–70.5)	169	71.9 (65.1–77.7)	85	37.2 (30.9–44.0)	69	29.0 (23.3–35.4)	156	68.4 (61.9–74.3)	162	70.5 (64.2–76.1)	178	78.4 (72.4–83.3)
Middle (n=108)	66	70.7 (61.5–78.6)	79	76.0 (66.2–83.7)	40	40.1 (30.7–50.2)	29	26.7 (19.2–35.8)	65	60.7 (50.7–69.9)	67	64.3 (55.3–72.4)	82	80.0 (70.4–87.1)
High (n=93)	58	65.1 (55.6–73.6)	70	79.6 (68.5–87.5)	36	42.5 (32.3–53.3)	23	23.8 (16.7–32.9)	57	65.8 (56.8–73.9)	54	60.5 (50.0–70.1)	71	82.8 (73.3–89.3)
P-value‡		0.50		0.40		0.69		0.63		0.36		0.17		0.70
NCES locale														
City (n=117)	81	79.7 (70.7–86.4)	87	74.9 (64.8–82.9)	50	44.3 (34.6–54.5)	47	39.9 (30.9–49.6)	70	62.5 (53.2–70.9)	68	59.1 (49.0–68.4)	86	78.7 (69.7–85.7)
Suburb (n=121)	88	78.4 (68.4–85.9)	95	79.7 (71.1–86.3)	50	42.9 (34.1–52.2)	39	30.0 (22.5–38.8)	84	67.4 (58.0–75.5)	84	67.6 (59.3–75.0)	97	83.4 (75.1–89.4)
Town (n=55)	27	49.2 (37.4–61.2)	38	72.5 (57.9–83.5)	17	37.9 (25.7–51.8)	15	28.6 (17.8–42.5)	32	64.7 (51.0–76.3)	31	59.9 (45.7–72.5)	36	69.0 (54.3–80.7)
Rural (n=102)	48	51.8 (42.2–61.2)	70	68.4 (57.6–77.5)	36	34.5 (25.4–44.9)	15	14.2 (8.6–22.5)	66	67.6 (58.0–75.9)	73	75.9 (66.3–83.5)	80	79.9 (70.5–86.8)
P-value‡		p<.001		0.33		0.48		p<.001		0.83		0.05		0.24
School poverty														
Low-poverty (n=77)	53	76.8 (64.7–85.7)	66	88.1 (77.4–94.1)	33	46.7 (34.9–58.8)	29	39.1 (28.0–51.4)	52	69.3 (57.1–79.3)	56	75.6 (63.5–84.6)	61	81.7 (70.0–89.6)
Mid-poverty (n=227)	118	57.4 (50.7–63.8)	159	71.0 (64.2–77.0)	73	33.2 (27.1–40.0)	51	21.4 (16.5–27.2)	139	62.8 (56.1–69.1)	142	64.0 (57.5–70.0)	167	76.9 (70.6–82.1)
High-poverty (n=96)	73	82.1 (71.3–89.4)	70	73.0 (61.8–81.9)	44	47.0 (36.5–57.8)	32	31.9 (22.8–42.6)	65	69.1 (57.9–78.5)	64	67.7 (56.4–77.3)	78	85.2 (75.5–91.5)
P-value‡		p<.001		0.02		0.03		0.009		0.48		0.24		0.26
Full time school nurse														
Yes (n=244)	159	69.1 (63.2–74.4)	186	75.3 (69.1–80.7)	93	39.9 (33.7–46.4)	70	26.5 (21.2–32.5)	150	61.3 (55.1–67.1)	161	65.9 (59.9–71.4)	184	77.4 (71.4–82.4)
No (n=179)	104	62.2 (54.5–69.4)	132	73.4 (65.7–79.9)	68	37.8 (30.6–45.6)	51	28.6 (22.4–35.7)	128	72.9 (65.7–79.1)	122	68.2 (60.7–74.9)	147	83.3 (76.9–88.2)

Characteristic	Required masks for students and staff		Inspected and validated existing HVAC systems		Replaced/upgraded HVAC		Installed or used HEPA filtration systems in classrooms		Opened doors when safe to do so		Opened windows when safe to do so		Adhered to at least to daily or between use cleaning schedules	
	n*	% (95% CI)†	n*	% (95% CI)†	n*	% (95% CI)†	n*	% (95% CI)†	n*	% (95% CI)†	n*	% (95% CI)†	n*	% (95% CI)†
P-value‡		0.16		0.67		0.68		0.64		0.01		0.62		0.15
School based health center														
Yes (n=69)	48	76.9 (64.6–85.9)	52	75.5 (62.6–85.0)	32	49.6 (36.9–62.3)	24	30.9 (20.6–43.6)	39	54.8 (42.1–66.8)	44	61.9 (48.9–73.3)	50	74.6 (62.2–84.0)
No (n=354)	215	64.2 (59.0–69.1)	266	74.4 (69.1–79.0)	129	36.8 (31.8–42.2)	97	26.5 (22.2–31.3)	239	68.2 (63.0–73.0)	239	67.8 (62.8–72.5)	281	80.8 (76.2–84.7)
P-value‡		0.06		0.85		0.06		0.47		0.04		0.36		0.26

CI = confidence interval; HEPA = high-efficiency particulate air; HVAC = heating, ventilation, and air conditioning; NCES = National Center for Education Statistics
*Unweighted numbers are presented.
†Weighted percents and 95% confidence intervals are presented.
‡Chi-square p-values are presented examining bivariate associations between each school-level characteristic and prevention strategy.

Appendix Table 4. Adjusted* odds ratios examining associations between school-level characteristics and school-level mask requirements, ventilation improvements, and cleaning procedures – National School COVID-19 Prevention Study, United States, October 5–November 19, 2021

Characteristic	Required masks for students and staff (n=344)	Inspected and validated existing HVAC systems (n=360)	Replaced/upgraded HVAC (n=360)	Installed or used HEPA filtration systems in classrooms (n=359)	Opened doors when safe to do so (n=360)	Opened windows when safe to do so (n=359)	Adhered to at least to daily or between use cleaning schedules (n=360)
	aOR (CI)a						
School level							
Elementary	1.08 (0.55–2.12)	0.56 (0.26–1.19)	0.66 (0.36–1.20)	1.27 (0.68–2.36)	0.98 (0.56–1.71)	1.40 (0.78–2.50)	0.66 (0.33–1.33)
Middle	1.19 (0.56–2.54)	0.70 (0.30–1.64)	0.86 (0.44–1.68)	1.12 (0.56–2.24)	0.68 (0.36–1.29)	0.97 (0.50–1.86)	0.85 (0.36–2.00)
High	Ref.	Ref.	Ref.	Ref.	Ref.	Ref.	Ref.
NCES locale							
City	Ref.	Ref.	Ref.	Ref.	Ref.	Ref.	Ref.
Suburb	1.40 (0.68–2.89)	1.29 (0.63–2.64)	1.10 (0.61–1.98)	0.71 (0.39–1.30)	1.45 (0.78–2.71)	1.58 (0.85–2.93)	1.76 (0.83–3.73)
Town	**0.38 (0.17–0.85)**†	1.02 (0.44–2.36)	1.01 (0.48–2.16)	0.83 (0.39–1.78)	1.60 (0.78–3.27)	1.55 (0.71–3.38)	0.69 (0.31–1.57)
Rural	0.65 (0.32–1.30)	0.93 (0.45–1.95)	0.99 (0.51–1.91)	**0.36 (0.17–0.76)**‡	**2.08 (1.03–4.17)**†	**4.51 (2.11–9.60)**§	1.14 (0.51–2.55)
School poverty							
Low-poverty	Ref.	Ref.	Ref.	Ref.	Ref.	Ref.	Ref.
Mid-poverty	0.57 (0.29–1.12)	**0.37 (0.16–0.84)**†	0.61 (0.33–1.12)	**0.52 (0.28–0.96)**†	0.75 (0.39–1.45)	**0.48 (0.24–0.95)**†	0.77 (0.35–1.68)
High-poverty	2.73 (0.98–7.59)	0.43 (0.17–1.08)	1.05 (0.53–2.08)	0.72 (0.35–1.50)	1.11 (0.50–2.47)	0.75 (0.32–1.74)	1.45 (0.58–3.62)
Full time school nurse							
Yes	1.21 (0.70–2.10)	1.11 (0.64–1.92)	0.93 (0.58–1.49)	0.85 (0.51–1.41)	**0.57 (0.34–0.96)**†	0.98 (0.59–1.62)	0.57 (0.31–1.06)
No	Ref.	Ref.	Ref.	Ref.	Ref.	Ref.	Ref.
School based health center							
Yes	1.67 (0.68–4.11)	1.14 (0.54–2.40)	1.57 (0.84–2.94)	1.30 (0.69–2.46)	0.69 (0.36–1.34)	0.92 (0.47–1.82)	0.65 (0.33–1.27)
No	Ref.	Ref.	Ref.	Ref.	Ref.	Ref.	Ref.

aOR = adjusted odds ratio; CI = confidence interval; HEPA = high-efficiency particulate air; HVAC = heating, ventilation, and air conditioning; NCES = National Center for Education Statistics
*For each COVID-19 prevention measure, models adjusted for school-level characteristics (school-based health center, school level, NCES locale, % of students eligible for free or reduced-price meals [FRPM], full-time school nurse) and the total number of new cases per 100,000 persons within the last 7 days in each county the school resides on September 23, 2021. Adjusted odds ratios, 95% confidence interval, and number of observations included are presented for each model.
†p<0.05, ‡p<0.01, §p<0.001; bolding indicates any finding that is significant at p<0.05.

Appendix Table 5. Prevalences and unadjusted associations between school-level characteristics and school-level physical distancing, isolation space, COVID-19 testing and screening, contact tracing, and quarantine protocols – National School COVID-19 Prevention Study, United States, October 5–November 19, 2021

	Maintained physical distance in classrooms		Had a school-based system to report COVID-19 outcomes		Had a COVID-19 isolation space in school		Offered COVID-19 diagnostic testing to students and staff		Offered COVID-19 screening testing to students and staff		Conducted contact tracing		Quarantined students identified as close contacts	
Characteristic	n*	% (95% CI)†	n*	% (95% CI)†	n*	% (95% CI)†	n*	% (95% CI)†	n*	% (95% CI)†	n*	% (95% CI)†	n*	% (95% CI)†
School level														
Elementary (n=236)	168	75.1 (69.1–80.3)	220	96.7 (93.4–98.4)	210	92.7 (88.4–95.5)	154	65.9 (58.9–72.2)	19	7.8 (5.0–11.8)	122	53.3 (46.4–60.0)	172	81.3 (75.3–86.2)
Middle (n=108)	77	73.5 (63.4–81.6)	96	92.7 (85.1–96.6)	96	93.8 (87.4–97.1)	79	75.9 (67.4–82.7)	13	12.0 (7.0–19.8)	55	51.9 (41.3–62.2)	81	87.1 (79.1–92.4)
High (n=93)	65	73.3 (62.7–81.7)	79	93.9 (86.7–97.4)	77	90.2 (80.3–95.4)	60	67.1 (54.5–77.7)	–‡	–‡	51	55.8 (45.9–65.3)	69	84.6 (73.5–91.6)
P-value¶		0.92		0.26		0.65		0.21		0.48		0.86		0.46
NCES locale														
City (n=117)	82	74.8 (65.2–82.4)	106	96.3 (90.6–98.6)	103	93.8 (87.3–97.1)	86	75.7 (65.9–83.3)	16	13.2 (8.2–20.5)	59	50.8 (41.0–60.6)	90	86.3 (77.5–92.0)
Suburb (n=121)	86	74.9 (65.7–82.3)	110	95.6 (89.5–98.2)	105	91.3 (84.0–95.5)	76	63.7 (54.2–72.2)	–‡	–‡	57	48.8 (39.2–58.5)	96	87.5 (79.2–92.8)
Town (n=55)	40	76.0 (62.8–85.6)	51	97.6 (84.0–99.7)	50	96.4 (85.2–99.2)	35	63.4 (46.7–77.4)	–‡	–‡	31	54.2 (40.1–67.7)	37	82.4 (67.6–91.3)
Rural (n=102)	74	73.2 (63.5–81.1)	90	91.6 (83.9–95.8)	89	91.2 (83.5–95.5)	72	73.9 (63.6–82.1)	–‡	–‡	55	57.1 (47.3–66.3)	73	81.2 (70.9–88.4)
P-value¶		0.98		0.35		0.62		0.18		0.46		0.66		0.63
School poverty														
Low-poverty (n=77)	53	72.5 (60.6–81.9)	73	100.0 (–)	65	89.3 (78.8–94.9)	47	63.2 (51.0–73.9)	–‡	–‡	44	59.4 (47.0–70.8)	60	87.0 (76.3–93.4)
Mid-poverty (n=227)	162	72.9 (66.5–78.6)	205	94.2 (90.1–96.6)	203	93.7 (89.7–96.2)	156	69.2 (62.4–75.2)	15	7.0 (4.2–11.6)	112	50.9 (43.9–57.8)	164	80.8 (74.4–85.9)
High-poverty (n=96)	71	77.8 (67.6–85.5)	86	94.4 (87.3–97.6)	87	93.7 (85.0–97.5)	64	68.0 (56.9–77.4)	15	15.2 (9.2–24.0)	52	53.8 (42.8–64.5)	76	89.9 (81.8–94.7)
P-value¶		0.66		0.12		0.47		0.66		0.09		0.49		0.11
Full time school nurse														

Characteristic	Maintained physical distance in classrooms		Had a school-based system to report COVID-19 outcomes		Had a COVID-19 isolation space in school		Offered COVID-19 diagnostic testing to students and staff		Offered COVID-19 screening testing to students and staff		Conducted contact tracing		Quarantined students identified as close contacts	
	n*	% (95% CI)†	n*	% (95% CI)†	n*	% (95% CI)†	n*	% (95% CI)†	n*	% (95% CI)†	n*	% (95% CI)†	n*	% (95% CI)†
Yes (n=244)	186	76.5 (70.4–81.7)	235	98.1 (95.5–99.2)	224	93.6 (89.6–96.1)	169	68.9 (62.3–74.8)	23	9.2 (6.1–13.6)	132	53.7 (47.1–60.2)	198	88.0 (82.8–91.8)
No (n=179)	124	71.0 (63.6–77.4)	160	90.5 (84.7–94.3)	159	90.7 (85.2–94.3)	124	68.6 (60.9–75.3)	17	9.5 (5.9–14.8)	96	53.0 (45.4–60.4)	124	76.6 (69.0–82.8)
P-value¶		0.23		p<.001		0.28		0.95		0.93		0.88		0.005
School based health center														
Yes (n=69)	52	74.4 (61.7–84.0)	63	94.3 (86.2–97.8)	64	94.4 (85.0–98.0)	52	75.5 (62.8–84.9)	–‡	–‡	40	58.3 (45.1–70.4)	56	89.5 (78.6–95.2)
No (n=354)	258	74.3 (69.4–78.7)	332	95.3 (92.3–97.1)	319	92.1 (88.6–94.5)	241	67.3 (61.9–72.4)	31	8.5 (6.0–11.8)	188	52.4 (46.9–57.9)	266	82.3 (77.5–86.3)
P-value¶		0.99		0.72		0.51		0.21		0.22		0.41		0.17

*Unweighted numbers are presented.
†Weighted percents and 95% confidence intervals are presented.
‡Estimate suppressed due to a relative standard error ≥30%.
¶Chi-square p-values are presented examining bivariate associations between each school-level characteristic and prevention strategy.

Appendix Table 6. Adjusted* odds ratios examining associations between school-level characteristics and school-level physical distancing, isolation space, COVID-19 testing and screening, contact tracing, and quarantine protocols – National School COVID-19 Prevention Study, United States, October 5–November 19, 2021

Characteristic	Maintained physical distance in classrooms (n=362)	Had a COVID-19 isolation space in school (n=357)	Offered COVID-19 diagnostic testing to students and staff (n=357)	Offered COVID-19 screening testing to students and staff (n=357)	Conducted contact tracing (n=357)	Quarantined students identified as close contacts (n=332)
	aOR (CI)					
School level						
Elementary	1.45 (0.77–2.74)	1.77 (0.56–5.60)	1.06 (0.53–2.09)	0.69 (0.27–1.73)	0.87 (0.51–1.50)	1.11 (0.45–2.75)
Middle	0.95 (0.46–1.95)	1.55 (0.45–5.29)	1.62 (0.74–3.56)	1.16 (0.40–3.35)	0.68 (0.36–1.30)	1.41 (0.50–3.96)
High	Ref.	Ref.	Ref.	Ref.	Ref.	Ref.
NCES locale						
City	Ref.	Ref.	Ref.	Ref.	Ref.	Ref.
Suburb	1.11 (0.58–2.16)	0.73 (0.25–2.18)	0.57 (0.30–1.10)	0.69 (0.27–1.74)	0.95 (0.53–1.71)	1.18 (0.48–2.89)
Town	1.11 (0.49–2.53)	5.44 (0.60–49.30)	0.57 (0.24–1.35)	0.93 (0.29–2.98)	1.23 (0.59–2.56)	0.68 (0.24–1.93)
Rural	1.12 (0.55–2.28)	0.75 (0.25–2.28)	0.94 (0.44–2.01)	0.70 (0.24–2.03)	1.30 (0.70–2.43)	0.86 (0.34–2.15)
School poverty						
Low-poverty	Ref.	Ref.	Ref.	Ref.	Ref.	Ref.
Mid-poverty	1.05 (0.54–2.01)	1.94 (0.72–5.21)	1.56 (0.83–2.95)	0.68 (0.25–1.89)	0.60 (0.33–1.11)	0.65 (0.28–1.56)
High-poverty	1.44 (0.65–3.17)	1.71 (0.55–5.27)	1.06 (0.51–2.21)	1.32 (0.49–3.60)	0.78 (0.39–1.57)	1.93 (0.61–6.06)
Full time school nurse						
Yes	1.37 (0.81–2.34)	1.55 (0.65–3.70)	0.95 (0.58–1.56)	0.89 (0.43–1.86)	1.09 (0.68–1.73)	**2.02 (1.05–3.91)†**
No	Ref.	Ref.	Ref.	Ref.	Ref.	Ref.
School based health center						

Characteristic	Maintained physical distance in classrooms (n=362)	Had a COVID-19 isolation space in school (n=357)	Offered COVID-19 diagnostic testing to students and staff (n=357)	Offered COVID-19 screening testing to students and staff (n=357)	Conducted contact tracing (n=357)	Quarantined students identified as close contacts (n=332)
	aOR (CI)					
Yes	1.06 (0.53–2.15)	1.23 (0.30–5.07)	1.59 (0.77–3.28)	1.49 (0.60–3.71)	1.11 (0.59–2.10)	1.97 (0.62–6.31)
No	Ref.	Ref.	Ref.	Ref.	Ref.	Ref.

aOR = adjusted odds ratio, CI = confidence interval, NCES = National Center for Education Statistics

*For each COVID-19 prevention measure, models adjusted for school-level characteristics (school-based health center, school level, NCES locale, % of students eligible for free or reduced-price meals [FRPM], full-time school nurse) and the total number of new cases per 100,000 persons within the last 7 days in each county the school resides on September 23, 2021. Adjusted odds ratios, 95% confidence interval, and number of observations included are presented for each model.

†p<0.05; bolding indicates any finding that is significant at p<0.05.

Appendix Table 7. Prevalences and unadjusted associations between school-level characteristics and school-level efforts to promote vaccination and track vaccination status of students and staff – National School COVID-19 Prevention Study, United States, October 5–November 19, 2021

	Provided information on COVID-19 vaccines to parents		Provided information on COVID-19 vaccines to students		Provided parents or students with information about catching up on missed healthcare (e.g., routine vaccines)		Provided COVID-19 vaccines on-campus to staff, students, or their families		Provided COVID-19 vaccines through school district events to staff, students, or their families		Tracked vaccination status of students		Tracked vaccination status of staff	
Characteristic	n*	% (95% CI)†	n*	% (95% CI)†	n*	% (95% CI)†	n*	% (95% CI)†	n*	% (95% CI)†	n*	% (95% CI)†	n*	% (95% CI)†
School level														
Elementary (n=236)	143	62.6 (55.6–69.0)	70	30.4 (24.5–37.0)	114	50.6 (43.6–57.6)	53	23.2 (18.0–29.5)	120	54.0 (47.4–60.4)	66	29.0 (23.2–35.6)	138	60.6 (54.2–66.7)
Middle (n=108)	67	65.9 (55.3–75.0)	54	50.7 (40.9–60.5)	56	56.3 (45.3–66.7)	37	37.9 (27.9–49.0)	49	46.3 (36.4–56.5)	33	33.3 (23.9–44.3)	53	53.9 (43.5–63.9)
High (n=93)	65	73.8 (62.3–82.7)	59	68.3 (56.8–77.9)	45	51.6 (39.2–63.8)	34	42.0 (32.0–52.7)	50	58.8 (46.3–70.3)	40	46.0 (35.3–57.1)	56	65.4 (54.0–75.2)
P-value‡		0.22		p<.001		0.7		0.002		0.26		0.03		0.29
NCES locale														
City (n=117)	81	72.1 (61.9–80.5)	51	48.0 (38.8–57.4)	65	59.5 (49.2–69.1)	32	30.8 (22.3–40.9)	64	59.1 (48.8–68.7)	35	30.8 (22.3–40.9)	77	71.8 (62.0–79.9)
Suburb (n=121)	81	69.5 (59.7–77.8)	45	38.6 (30.4–47.5)	57	50.0 (39.9–60.1)	37	34.1 (25.3–44.3)	68	60.2 (51.0–68.7)	34	30.4 (22.1–40.2)	74	63.3 (53.4–72.2)
Town (n=55)	32	63.0 (48.9–75.2)	22	43.7 (32.4–55.7)	29	56.0 (39.6–71.2)	18	36.7 (26.1–48.7)	26	45.8 (30.7–61.8)	24	50.6 (36.7–64.3)	31	61.4 (47.5–73.7)
Rural (n=102)	57	58.3 (47.6–68.4)	46	45.8 (35.6–56.2)	46	46.8 (36.3–57.7)	24	24.1 (16.2–34.3)	39	41.8 (32.0–52.4)	31	31.3 (22.2–42.0)	40	41.1 (31.5–51.5)
P-value‡		0.20		0.48		0.36		0.34		0.03		0.08		p<.001
School poverty														
Low-poverty (n=77)	51	69.2 (56.8–79.4)	24	30.5 (20.6–42.7)	30	41.6 (30.2–54.0)	16	21.2 (12.9–32.7)	32	46.2 (34.5–58.3)	29	41.5 (30.1–53.9)	47	66.0 (53.8–76.4)
Mid-poverty (n=227)	128	59.4 (52.3–66.1)	84	39.2 (32.7–46.0)	118	55.2 (47.9–62.3)	62	30.5 (24.3–37.5)	111	50.4 (43.7–57.1)	72	32.9 (26.5–40.1)	117	54.1 (47.2–61.0)
High-poverty (n=96)	71	77.1 (66.6–85.1)	55	60.3 (49.3–70.4)	51	54.3 (43.1–65.0)	31	32.5 (23.1–43.5)	58	65.5 (54.7–74.9)	25	28.5 (19.4–39.8)	57	63.9 (53.2–73.4)
P-value‡		0.01		p<.001		0.16		0.29		0.03		0.28		0.12
Full time school nurse														
Yes (n=244)	162	65.9 (59.2–72)	111	44.8 (38.7–51)	135	56.7 (49.7–63.5)	82	36.6 (30.4–43.2)	136	55.7 (49–62.2)	93	39.2 (32.9–45.8)	152	63.6 (57.1–69.7)

Characteristic	Provided information on COVID-19 vaccines to parents		Provided information on COVID-19 vaccines to students		Provided parents or students with information about catching up on missed healthcare (e.g., routine vaccines)		Provided COVID-19 vaccines on-campus to staff, students, or their families		Provided COVID-19 vaccines through school district events to staff, students, or their families		Tracked vaccination status of students		Tracked vaccination status of staff	
	n*	% (95% CI)†	n*	% (95% CI)†	n*	% (95% CI)†	n*	% (95% CI)†	n*	% (95% CI)†	n*	% (95% CI)†	n*	% (95% CI)†
No (n=179)	113	65.6 (57.8–72.7)	72	41.8 (34.3–49.6)	80	45.5 (37.7–53.5)	42	22.3 (16.5–29.5)	83	48.9 (41.1–56.8)	46	25.3 (19.2–32.6)	95	54.2 (46.4–61.7)
P-value‡		0.96		0.55		0.03		0.003		0.20		0.004		0.07
School based health center														
Yes (n=69)	50	73.9 (60.8–83.8)	38	56.6 (43.4–69)	40	61.6 (48.4–73.3)	33	49.4 (36.6–62.3)	47	71.3 (58.3–81.5)	29	41.7 (29.7–54.8)	49	73.8 (60.9–83.6)
No (n=354)	225	64.1 (58.5–69.4)	145	40.9 (35.7–46.3)	175	50.4 (44.6–56.1)	91	27.2 (22.4–32.6)	172	49.3 (43.9–54.8)	110	32.0 (27–37.5)	198	57.1 (51.7–62.3)
P-value‡		0.15		0.03		0.11		p<.001		0.002		0.15		0.01

CI = confidence interval; HEPA = high-efficiency particulate air; HVAC = heating, ventilation, and air conditioning; NCES = National Center for Education Statistics

*Unweighted numbers are presented.

†Weighted percents and 95% confidence intervals are presented.

‡Chi-square p-values are presented examining bivariate associations between each school-level characteristic and prevention strategy.

Appendix Table 8. Adjusted* odds ratios examining associations between school-level characteristics and school-level efforts to promote vaccination and track vaccination status of students and staff – National School COVID-19 Prevention Study, United States, October 5–November 19, 2021

Characteristic	Provided information on COVID-19 vaccines to parents (n=355)	Provided information on COVID-19 vaccines to students (n=355)	Provided parents or students with information about catching up on missed healthcare (e.g., routine vaccines) (n=355)	Provided COVID-19 vaccines on-campus to staff, students, or their families (n=355)	Provided COVID-19 vaccines through school district events to staff, students, or their families (n=355)	Tracked vaccination status of students (n=355)	Tracked vaccination status of staff (n=355)
	aOR (CI)						
School level							
Elementary	**0.49 (0.25–0.97)†**	**0.15 (0.08–0.29)§**	1.13 (0.59–2.14)	**0.47 (0.26–0.87)†**	0.63 (0.33–1.20)	**0.45 (0.24–0.83)†**	0.67 (0.34–1.31)
Middle	0.58 (0.26–1.25)	**0.39 (0.20–0.79)‡**	1.32 (0.63–2.78)	0.76 (0.37–1.57)	**0.44 (0.21–0.92)†**	0.50 (0.23–1.07)	**0.44 (0.20–0.95)†**
High	Ref.	Ref.	Ref.	Ref.	Ref.	Ref.	Ref.
NCES locale							
City	Ref.	Ref.	Ref.	Ref.	Ref.	Ref.	Ref.
Suburb	1.18 (0.60–2.30)	1.04 (0.57–1.91)	0.73 (0.39–1.34)	1.69 (0.84–3.40)	1.29 (0.71–2.33)	0.88 (0.45–1.74)	0.78 (0.41–1.49)
Town	0.89 (0.40–2.00)	1.02 (0.50–2.12)	0.98 (0.42–2.32)	1.66 (0.81–3.41)	0.52 (0.24–1.15)	**3.09 (1.36–7.01)‡**	0.79 (0.36–1.72)
Rural	0.79 (0.39–1.61)	1.31 (0.65–2.66)	0.67 (0.34–1.31)	1.05 (0.49–2.27)	**0.45 (0.23–0.88)†**	1.28 (0.62–2.67)	**0.45 (0.23–0.90)†**
School poverty							
Low-poverty	Ref.	Ref.	Ref.	Ref.	Ref.	Ref.	Ref.
Mid-poverty	0.65 (0.34–1.25)	1.27 (0.65–2.48)	**1.91 (1.06–3.44)†**	1.70 (0.82–3.54)	1.43 (0.80–2.57)	0.67 (0.35–1.30)	0.85 (0.46–1.57)
High-poverty	1.40 (0.65–3.02)	**3.88 (1.81–8.30)§**	1.71 (0.84–3.47)	2.13 (0.93–4.88)	**2.47 (1.23–4.98)†**	0.60 (0.28–1.28)	1.34 (0.62–2.90)
Full time school nurse							
Yes	0.78 (0.47–1.30)	0.85 (0.52–1.41)	1.18 (0.73–1.91)	1.69 (0.98–2.91)	1.01 (0.63–1.63)	**1.80 (1.07–3.03)†**	1.50 (0.92–2.45)
No	Ref.	Ref.	Ref.	Ref.	Ref.	Ref.	Ref.
School based health center							

Characteristic	Provided information on COVID-19 vaccines to parents (n=355)	Provided information on COVID-19 vaccines to students (n=355)	Provided parents or students with information about catching up on missed healthcare (e.g., routine vaccines) (n=355)	Provided COVID-19 vaccines on-campus to staff, students, or their families (n=355)	Provided COVID-19 vaccines through school district events to staff, students, or their families (n=355)	Tracked vaccination status of students (n=355)	Tracked vaccination status of staff (n=355)
				aOR (CI)			
Yes	1.40 (0.68–2.87)	1.27 (0.67–2.40)	1.35 (0.72–2.54)	**2.00 (1.03–3.89)**[†]	**2.25 (1.18–4.30)**[†]	1.25 (0.67–2.34)	1.87 (0.87–3.99)
No	Ref.	Ref.	Ref.	Ref.	Ref.	Ref.	Ref.

aOR = adjusted odds ratio, CI = confidence interval, NCES = National Center for Education Statistics

[*]For each COVID-19 prevention measure, models adjusted for school-level characteristics (school-based health center, school level, NCES locale, % of students eligible for free or reduced-price meals [FRPM], full-time school nurse) and the total number of new cases per 100,000 persons within the last 7 days in each county the school resides on September 23, 2021. Adjusted odds ratios, 95% confidence interval, and number of observations included are presented for each model.

[†]p<0.05, [‡]p<0.01, [§]p<0.001; bolding indicates any finding that is significant at p<0.05
